# Toward *In Silico* Design of Protein–Protein Interaction Stabilizers

**DOI:** 10.1021/acscentsci.3c00545

**Published:** 2023-05-09

**Authors:** Jintao Zhu, Luhua Lai, Jianfeng Pei

**Affiliations:** † Center for Quantitative Biology, AAIS, Peking University, Beijing, 100871, China; ‡ Peking-Tsinghua Center for Life Sciences, AAIS, Peking University, Beijing, 100871, China; § BNLMS, College of Chemistry and Molecular Engineering, Peking University, Beijing, 100871, China

**Figure 1:**
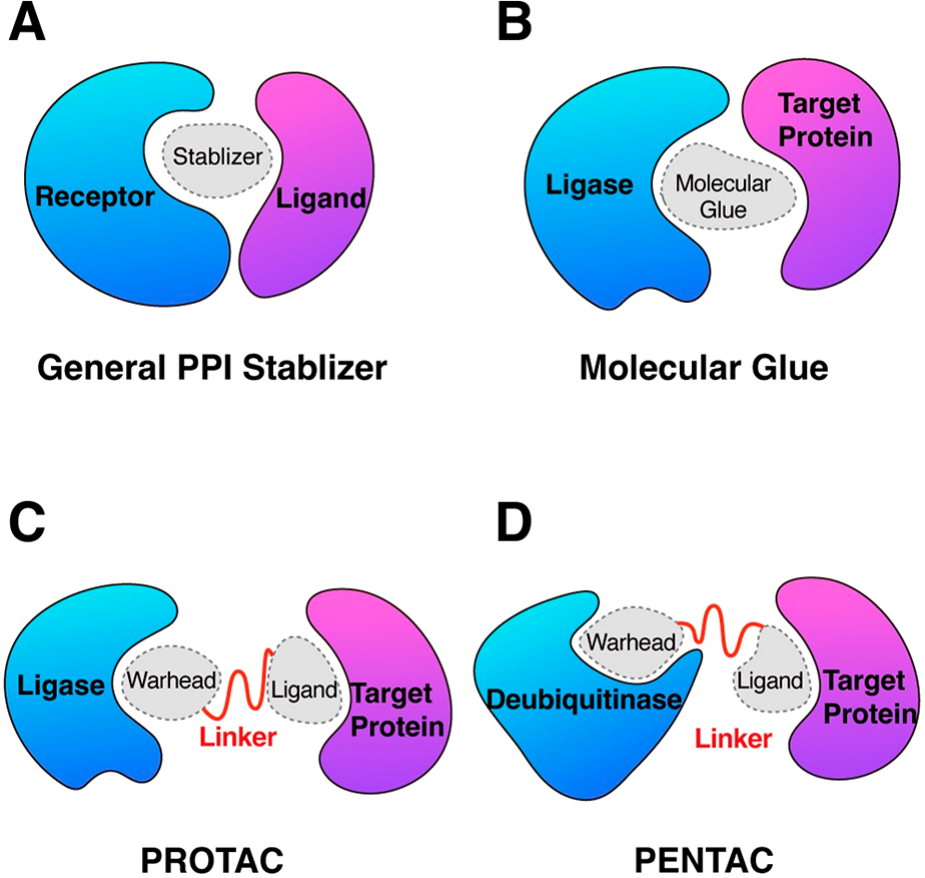
Four types of popularly studied PPI stabilizers. (A) General PPI stabilizer which stabilizes receptor–ligand binding directly or allosterically. (B) Molecular glue which induces or enhances the binding of E3 ligase and the target protein. (C) Proteolysis targeting chimera (PROTAC) which induces or enhances the binding of E3 ligase and the protein of interest (target protein). (D) What is referred to here as protein enhancement targeting chimera (PENTAC) is a PPI stabilizer that stabilizes the binding of deubiquitinase and the target protein. (B), (C), and (D) are subsets of (A).

Modulation of protein–protein interactions (PPIs) by small molecules is an emerging and highly promising approach for next-generation drug discovery. Compared to much studied PPI inhibitors, PPI stabilizers possess unique advantages due to their uncompetitive nature and potentially high specificity.^1^Figure 1 shows four types of popularly studied PPI stabilizers. Although there are examples of PPI stabilizers in clinic use, such as the immunosuppressant cyclosporine and the immunomodulatory imide drugs (IMiDs) thalidomide and lenalidomide, the majority of reported PPI stabilizers are primarily serendipitous,^2,3^ and there are currently no *in silico* rational design methods to empower the discovery of PPI stabilizers. To promote rational design of PPI stabilizers, the mechanism of action behind the receptor-stabilizer-ligand ternary complex (RLS) and how such interface-binding small molecules can be discovered should be answered first. In this issue of *ACS Central Science*, Martin Zacharias and Shu-Yu Chen proposed a dual-binding mechanism and computational design principle to screen and optimize potential PPI stabilizers.^4^ They proposed that a similar stabilizer interaction strength with each protein partner is an important prerequisite for effective stabilization, which is unrelated to the total interaction free energy between the receptor–ligand complex and the stabilizer.

*In silico* structure-based PPI stabilizer design is more challenging than the conventional binary target-ligand system. One consideration is the stabilization efficiency of the ternary complex which impacts downstream biological function intervention. To develop a computational method and accelerate rational PPI stabilizer design, Martin Zacharias and Shu-Yu Chen systematically investigated 18 RLS crystal structures from an energetic perspective, including diverse stabilizer-induced and stabilizer-enhanced PPI complexes. Using buried surface area analysis and further interaction free energy calculation using MD simulations and MM/GBSA, they found that for most cases stabilizers tend to have similar contacts with the two partners. Stabilizers that do not conform to this rule may follow an allosteric mechanism. They also revealed that potent stabilizers tend to have more favorable interactions with their weaker binding partner. However, no correlation between the stabilizing potency and the total energy of interaction was found. This interesting finding would be helpful to guide PPI stabilizer screening and optimization. The authors also suggest a hypothesis that PPI inducers tend to shield the unfavorable RL interactions and keep the favorable ones, while PPI enhancers either preserve or augment the pre-existing favorable contacts. The exposed unfavorable contact residues hinder RL binding, resulting in nondetectable or weak affinity between the stabilizer-induced PPI partners. Overall, PPI stabilizers could be designed with strong binding affinity to the weaker stabilizer-binding partner and a similar magnitude of interaction free energy.

Before the above principle is applied to real-world PPI stabilizer design, another consideration is druggable pocket prediction on the RL binding interface. Notably, in most cases only stabilizer-free (modeled) RL complexes are available, and it is difficult to detect pockets in the mostly flat interfaces. Obtaining an accurate ternary complex by inducing the stabilizer-bound pocket could prove to be a formidable challenge, necessitating considerable exertion. Interestingly, the authors found pocket detecting methods like Fpocket 4.0^5^ could be used to detect stabilizer-free RL complex interface pockets, and short MD simulations were sufficient to reveal cryptic stabilizer-binding pockets. However, the authors mainly focused on well-defined PPI complexes that may lead to overoptimistic results. In fact, how to model the accurate ternary complex remains largely underexplored.

Combining pocket probing, molecular docking, and MD simulations, the authors developed a general protocol for PPI stabilizer discovery. They demonstrated that the dual binding mechanism can be helpful to improve the success rate of identifying potent stabilizers. They validated this hypothesis using 13 potential 13-4-4/ChREBP stabilizers reported by Christian Ottmann and co-workers,^6^ where the two most potent stabilizers have measured EC_50_. Based on the dual binding mechanism, the two stabilizers could be successfully ranked at the top. This work has taken the first step toward computer-aided discovery of PPI stabilizers; however, further wet-experimental validation is required to confirm the theoretical hypothesis.

One of the most active fields for PPI stabilization is molecular glue (MG)-mediated targeted protein degradation through inducing or strengthening the engagement between the E3 ubiquitin ligase and neosubstrate (Figure 1B).^2^ The contribution of Martin Zacharias and Shu-Yu Chen provides a novel approach to assist the development and optimization of MGs. However, there are still urgent needs in computational modeling-assisted MG development. In the accompanying articles of this paper, there are two reports^7,8^ that develop MGs based on experimental screening and design. Recently, Wang and co-workers presented PPI-Miner to search potential protein interacting partners utilizing protein structure motif and sequence motif matching.^9^ They searched 1739 potential neosubstrates for cereblon (CRBN) E3 ligase, and 16 of them had been experimentally validated by previous studies. The dual-binding protocol may not only assist the optimization of known active MGs, but also design novel MGs targeting neosubstrate or new E3 ligase. Although there are concerns that cellular degradation may not only depend on the affinity of MGs in the ternary complex, a recent study on cereblon E3 ligase modulators (CELMoDs) shows that the predicted binding affinity given by MM/GBSA also has a good correlation with downstream degradation.^10^ We envisage that with the accumulation of data, machine learning or deep learning will further help prioritize PPI stabilizer-like features. Advanced *in silico* modeling methods can be useful for understanding pocket induction and the ternary complex formation mechanism. In conclusion, this work opens up a new avenue in *in silico* modeling, virtual screening and design of extensive protein–protein stabilization, especially for MG design, significantly extending the druggable genome.
